# Ultrasound Intra Body Multi Node Communication System for Bioelectronic Medicine

**DOI:** 10.3390/s20010031

**Published:** 2019-12-19

**Authors:** Banafsaj Jaafar, Junwen Luo, Dimitrios Firfilionis, Ahmed Soltan, Jeff Neasham, Patrick Degenaar

**Affiliations:** 1 School of Engineering, Newcastle University, Newcastle upon Tyne NE1 7RU, UK; b.j.rasool2@newcastle.ac.uk (B.J.); dimitrios.firfilionis@newcastle.ac.uk (D.F.); jeff.neasham@newcastle.ac.uk (J.N.); 2 Computing technology lab, DAMO academy, Alibaba Group, Hangzhou 310030, China; junwen.luo@alibaba-inc.com; 3 NISC Research group, Nile University, Sheikh Zayed 12677, Egypt; asoltan@nu.edu.eg

**Keywords:** ultrasound transducer, throughput, biomedical, intra body communication, bioelectronic nodes, bit error rate, packet error rate, communication protocol

## Abstract

The coming years may see the advent of distributed implantable devices to support bioelectronic medicinal treatments. Communication between implantable components and between deep implants and the outside world can be challenging. Percutaneous wired connectivity is undesirable and both radiofrequency and optical methods are limited by tissue absorption and power safety limits. As such, there is a significant potential niche for ultrasound communications in this domain. In this paper, we present the design and testing of a reliable and efficient ultrasonic communication telemetry scheme using piezoelectric transducers that operate at 320 kHz frequency. A key challenge results from the multi-propagation path effect. Therefore, we present a method, using short pulse sequences with relaxation intervals. To counter an increasing bit, and thus packet, error rate with distance, we have incorporated an error correction encoding scheme. We then demonstrate how the communication scheme can scale to a network of implantable devices. We demonstrate that we can achieve an effective, error-free, data rate of 0.6 kbps, which is sufficient for low data rate bioelectronic medicine applications. Transmission can be achieved at an energy cost of 642 nJ per bit data packet using on/off power cycling in the electronics.

## 1. Introduction

Bioelectronic medicine is potentially a significant new therapeutic contribution to the treatment of chronic disease. The basic concept (also sometimes referred to as electroceuticals) is to stimulate the body’s organs via the autonomic nervous system to modulate the body’s biochemistry and thus treat disease. If the modulation can be closer to the required biochemistry and better match the body’s rhythms, then it might prove superior to pharmaceutical treatments for certain chronic disease conditions [1]. Therefore, a significant research effort is being undertaken in the bioelectronics domain. The key target is for those with chronic conditions such as diabetes [2], inflammatory bowel disease [3], lupus [4] and arthritis [5] and other age-related conditions. In the longer term, bioelectronic therapies may help with healthy aging, even in relatively athletically active individuals [6].

The vagus (or 10th cranial) nerve has been proposed as the primary stimulus point for bioelectronic medicine. The vagus nerve controls a significant proportion of the parasympathetic nervous system. Stimulation of this nerve can, therefore, result in modulating the activity of downstream organs to achieve therapy. However, an important development is to progress from open-loop, pacemaker type, stimuli to closed-loop control methodologies which modify therapeutic stimuli according to need. Such systems will therefore also need sensors from perhaps downstream organs which can provide physiological information. Examples include blood oxygen and glucose levels, heart rate, chemical sensing, mechanical motion, and bioelectronic activity [7,8].

**Figure 1:**
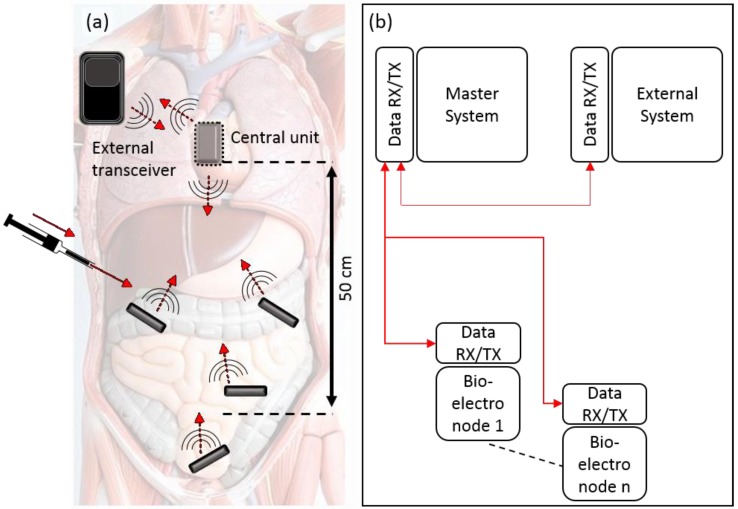
(**a**) Concept image of how the implants can be embedded inside the human body and scale down to be injectable; (**b**) The slave system bioelectronic nodes will be capable of collecting the sensed data and transmit the information to the central unit (master) that embedded inside the chest. The latter will communicate with the external transceiver.

Bioelectronic therapies would need to comprise an active implant (Active Implantable Medical Device or AIMD) which can gather information, perform processing and determine stimulus. As downstream organs are geographically separated by tens of centimeters, a possible architecture is for a central implant unit together with satellite units called bioelectronic nodes (BeNs) which provide sensing and perhaps stimulation. This configuration can be seen in Figure 1. Ideally, such bioelectronic nodes would be injected to their target locations rather than be surgically inserted. As such, the devices would need to be in the mm size range. Such devices would have small batteries and thus be limited in operation. i.e., duty cycles would be for a few milliseconds each hour or day. However, such operation is sufficient for bioelectronic therapies which only need to infrequently monitor physiological responses.

Short term percutaneous power and data cabling can be acceptable for implantable systems that are utilized for days or perhaps a few weeks. However, a break in the skin barrier for longer than that presents an infection risk. As such, the most common form of transcutaneous communication—i.e., between outside and inside the body is via radio frequency (RF) methods. Such communication must adhere to MedRadio/ISM bands such as (402–405 MHz), (902–928 MHz) and 2.4 GHz. However, the body’s absorption of radio waves increases with frequency [9,10]. At lower frequencies in the kHz range, such as that used in sacral stimulators, deep penetration can be used. However, efficient antennae scale inversely with frequency and can be very large in the cm-scale in the kHz range [11]. This is not desirable for long term mm size bioelectronic implants. Furthermore, for an arrangement as per Figure 1, intrabody communication is required between a master system and bioelectronics nodes. Transmission distances between these devices can be in the tens of centimeters, making optical and near field RF methods unsuitable.

Ultrasonic waves, in contrast, can traverse tissues with an attenuation coefficient of 0.6 dB/cm at 1 MHz. In addition, the speed of sound in saline is ~1540 m/s. For frequencies between 100 kHz and 1 MHz, wavelengths are between 1.5–15 mm leading to small transducers [12]. As such, ultrasound communications are highly suited to the task of communication with deep tissue implants to either the outside world or to disperse bioelectronics nodes.

Ultrasound backscattering communication technology has previously been demonstrated in [13] by Ghanbari et al. They presented two main devices: the first device is the implanted neural recording that operates when receiving a high transmission power pulses from the second device that called the interrogator which is non-invasively placed on the tissue surface. The recorded data will be modulated on the reflected signal using impedance modulation. However, this architecture has some disadvantages. It suffers from backscatter temporal jitter that affects the recovered information and it requires a high signal to noise ratio (SNR) to operate properly. Nevertheless, for non-safety critical operations, it can adequately be used. In contrast, for therapies requiring error-free (safe) intervention, the lack of any forward error correction or detection techniques can present a problem.

The transmission bandwidth is another critical merit in communication. Fundamentally, the low operating frequency of the ultrasound transducer and limited bandwidth drastically affect the achievable data rate and limit the information that can be transmitted. Moreover, it is difficult to increase the communication center frequency significantly beyond 1 MHz due to increasing attenuation [14,15]. To achieve reliable and sufficient communication, modern modulation can be utilized such as quadrature phase-shift keying and quadrature amplitude modulation [16]. However, such a modulation required more complex hardware and software hence energy-hungry receiver which will increase power and the bioelectronic device size. Therefore, in our system, we employed ON-OFF keying (OOK) that requires a less complex receiver in terms of hardware and processing.

An experimental demonstration of OOK-ultrasound communications was carried out by Kondapalli et al. [17] using a chicken tissue phantom. They examined communication distances up to 3 cm using an omnidirectional commercial ultrasound crystal of 1 mm diameter at frequency 1.3 MHz and a transmission voltage of up to 20 Vp-p. The team achieved a bit error rate (BER) of $10^{-2}$ and a data rate of 2.5 kbps without using error correction code. Wang et al. [16] explored directional ultrasonic communications using mineral oil to mimic the human body. They utilized a plate ultrasound transducer of dimensions 1.08 × 1.08 × 1.44 ${\mathrm{mm}}^{3}$ at 1 MHz, and a transmission voltage of up to 10 Vp-p. At 5 cm distance between the transmitter and receiver, the team achieved a BER less than $\;10^{-4}$, resulting in a data rate of 125 kbps.

Another study carried out by Santagati et al. [18] which achieved a high data rate of 70 kbps and BER less than $10^{-6}$. However, the commercial ultrasound transducer that has been used was directional with 9.5 mm diameter and the resonance frequency was high 5 MHz, which could have significant attenuation problems through deep tissue. Finally, Weber et al. [19] utilized a plate shape 1.8 × 1.08 × 1.08 ${\mathrm{mm}}^{3}$ piezo transducer at a carrier frequency of 790 kHz, using castor oil to mimic the human body. Over a 12 cm distance, they achieved a high signal to noise ratio of 29 dB, and thus a BER of less than 10^−5^ using OOK modulation.

From these past efforts, plate and disc shapes ultrasound transducers are more efficient for point to point data communication over short distances. However, over longer distances, if the plates become directionally misaligned, then the communication would become problematic. In such instances, non-directional transmitters and receivers would be a more efficient method to achieve error-free data.

This paper presents a design and testing of reliable and efficient ultrasonic communication telemetry using omnidirectional transducers to implement intra-body communication inside the human body. We implement a prototype to evaluate the system performance in saline and up to 30 cm distance between the transmitter and receiver. Short pulses sequences with guard intervals are employed to minimize the multipath effect. A Reed-Solomon error correction coding scheme has been employed to achieve reliable communication at low SNR (at longer distances). Energy per bit and current consumption for the transmitter and receiver main parts are presented and discussed in terms of battery life.

## 2. Ultrasound Transduction

Ultrasonic communication requires transducers to convert electrical signals into ultrasonic waves. Ultrasound transducers have previously been manufactured in different shapes such as discs [20], plates [21,22] and tubes [23] based on the application and operation mode. Disc and plate shapes provide directional beams and are thus efficient at the point to point transmission. However, in some instances line of sight may not always be possible. Furthermore, over time, implants can move and rotate. As such, directional shapes could prove to be inefficient for communication between bioelectronic implant devices [24]. An alternative, therefore, is to utilize inherently omnidirectional transducers-i.e., those that emit a pressure wave equally in all directions.

The capacitive micromachined ultrasound transducer (CMUT) is one ultrasound transduction device that has been used for ultrasound powering and imaging. Those devices are MEMS-based structures, each device in the CMUT array consists of a parallel plate capacitor, the first plate is fixed in the silicon substrate and the second plate is propped by a flexible membrane. However, investigations of these devices have been limited as they require high DC bias voltages such as 40 V [25] and 16 V [26]. High voltages requirement makes the operating architecture more complicated. Furthermore, the wave propagation is directional which makes it not suitable to be utilized in multipoint (omnidirectional) communication scenarios. Moreover, squeeze film damping effects and the requirement for vacuum-sealing can result in added mechanical noise in CMUT arrays [27,28].

An alternative is to use lead zirconate titanate (Pb[Zr_X_Ti_1-X_]O_3_: PZT) piezo-transducers in a tube architecture. These have been shown to have the most efficient piezoelectrical properties in terms of sensitivity. PZT transducers are 10 dB more sensitive compared to CMUT [29]. However, it should be noted that although PZT is currently the most efficient of the current batch of piezo transduces, 60% of the material is lead oxide [30], which is a toxic heavy metal, particularly to the nervous system. Nevertheless, we utilized the material as it is currently best in class, and this paper primarily focusses on the communication system as a whole. We would first point out that any device would be encapsulated and thus would be safe at least for short periods and is thus suitable for in-vivo research, which would need to be performed before any clinical translation. Nevertheless, further into the future, the toxicity of PZT will mean that alternative (lead/heavy metal free) alternatives such as potassium sodium niobite [31] will need to be explored.

**Figure 2:**
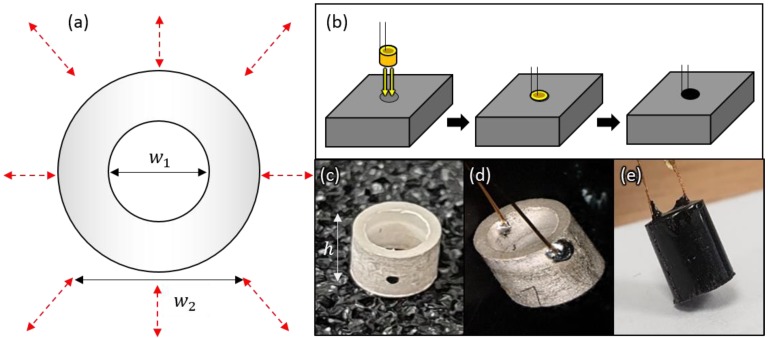
The omnidirectional ultrasound transducer (**a**) Radial mode vibration of the active element (top view), dimensions: $w_{1}\;$= 2.5 mm,$\;w_{2}\;$ = 3.5 mm (**b**) Steps that used to Implement and encapsulate the transducer, Assembly the active elements on the CNC machined metal mold. Then, the active element aligned in the centre of the mold with 1 mm gap and the set is filled with polyurethane; (**c**) Bare piezoelectric crystal of height h = 3 mm; (**d**) Wire bonding the inner and outer diameter electrode; (**e**) The transducer final shape after curing.

A tube shaped PZT can be operated efficiently in the omnidirectional mode [23], as per Figure 2a. As a result, it is more suitable for the multipoint ultrasonic communication scheme system presented in Figure 1. We utilized off-chip piezoelectric tube transducers, as shown in Figure 2a, of dimensions (h = 3 mm, $w_{1}$= 2.5 mm and $w_{2}$ = 3.5 mm) with 290 kHz radial resonant frequency are implemented. We purchased the bare piezo-transducers from Meggitt A/S (Kvistgaard, Denmark), we specifically utilized soft Pz27 (Navy II) material as it has good coupling factors (Kp) of 0.59, high charge coefficients (d33) of 440 pC/N, high Curie temperature of 350 °C and suitable for medical application [32]. The resonance frequency ($f_{r}$) is calculated as shown in the following Equation (1):

(1) $$f_{r}=\frac{2\times N_{md}}{w_{1}+w_{2}}$$

where $N_{md}$ is the speed of the sound of Pz27 (medium diameter speed) which is 880 m/s [32]. $w_{1}\;and\;w_{2}$ are the outer and inner diameters respectively.

Encapsulation of transducers and electronics is important in order to insulate them from the surrounding environment (saline/tissue), achieve mechanical robustness and to achieve wide communication bandwidth. We utilized polyurethane (PU) as a coating material as its acoustic impedance is 1.8 MRayls which is close to that of water (1.5 MRayls). Such impedance matching is important in minimizing possible reflections at the interface. Also, PU has excellent water resistance [33], and has already been used in the pacemaker leads from a Medtronic model P1501DR [34], indicating biocompatibility. We specifically utilized a black polyurethane resin (UR5041) from Electrolube (Leicestershire, UK).

The target encapsulation layer thickness is 0.5 mm for the surrounded diameter and fully inserted into the inner diameter. Therefore, a custom mold was designed to fulfill the aforementioned requirement. The ultrasound transducer was fixed inside the mold making sure it was centered and 0.5 mm space between the mold and outer diameter. Figure 2b shows the final step after injecting the PU to the mold, then baked in the oven for 24 h at 40 °C. Figure 2c shows the tube shape bare PZT crystal with silver electrodes on the curved flat surfaces and black single dot to indicate polarization. To achieve a better solder connection to the electrode surface, we scraped the surface of the crystal with emery paper, in order to remove the layer of silver oxide. We thereby attained a bright and flat surface of the electrode. Afterward, the surface of the electrode was prepared by swabbing the surface with isopropyl alcohol (IPA) to remove any grease or dust. Low melting temperature solder 180 °C from Henkel technologies (Hertfordshire, UK), 0.46 mm thickness in order to avoid the chance of excessive heating that may damage the silver electrode. A small amount of solder was applied in the desired location (near the edge of inner and outer diameters of the PZT crystal), then Unistrand Enamel ultrafine copper wire of diameter 0.13 mm from Rapid (Essex, UK) is connected to the crystal taking into account not moving the wire prior cool down the joint, Figure 2d illustrates the PZT crystal with electrical leads connected. The final encapsulated transducer is shown in Figure 2e.

## 3. System Architecture

**Figure 3:**
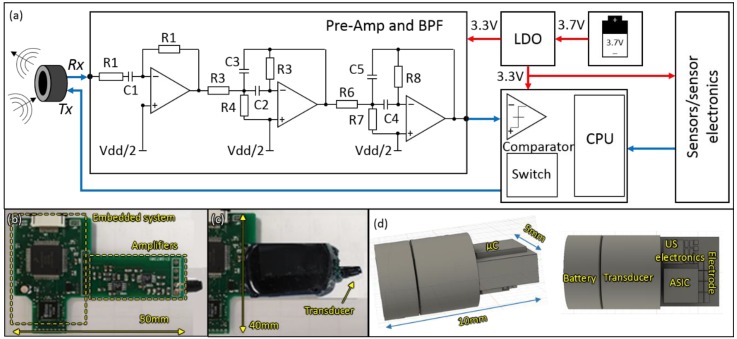
Block diagram of the whole system showing the three main parts of the implant: (**a**) The one directional ultrasound communication link that operates at 320 kHz resonance frequency and driven by three stages filters and amplifier, the embedded control system which digitizes and process the data, and the sensors unit; (**b**) and (**c**) The designed PCB for overall system with (50 × 40) mm dimensions, wire bonding the transducers to the PCB; (**d**) 3D model of the system before the passivation that shows the main utilized components and how it can be miniaturized to be injectable.

In order to explore the fundamental communication architecture, we developed a benchtop system that can be scaled considerably if all implemented onto a monolithic microelectronic unit. Its key components are illustrated in Figure 3.

The test system comprises three main units: (i) the ultrasound communication link, (ii) the embedded control unit, and (iii) the sensor unit. In the case of (i), the receiver link is driven by the ultrasound electronics that include filtration and amplification stages. As per above, the purpose here is to explore the optimal communication link, so these have been implemented using discrete amplifiers. Furthermore, the microcontroller and sensor electronics are also discrete as per Figure 3b,c. In a future/final micro-system, we would envisage much of the electronics to compress to a single application-specific integrated circuit (ASIC) for sensing and communications and perhaps a separate microcontroller chip for processing. We would also use a custom ceramic base which would be more suitable for implantable devices compared to a PCB base. A concept final configuration can be seen in Figure 3d.

### 3.1. Ultrasound Communication Link

The transmitter operates with pulse modulation from the microcontroller described in Figure 3. Pulses with a fundamental frequency of 320 kHz are passed from the microcontroller to the transmitter transducer. The pulses have a voltage of 3.3 V which is sufficient to vibrate the transducer, generating an omnidirectional pressure wave at its resonance frequency.

On the receiver side, amplifier circuitry is required to increase the signal sufficiently to be detected by a threshold detector on the microcontroller. It must also provide a tight filter around the 320 kHz transmission band to maximize signal to noise ratio. The acoustic signal is very weak, typically in the µV as measured by past literature [35] and our own measurements. An amplifier system is therefore required with a gain of 35–40 dB to match a threshold detector, which requires a minimum of 23 mV root mean square (RMS) on the microcontroller. 

To ensure maximum signal to noise performance, we developed a 4th order cascaded multiple feedback bandpass filter, preceded by a broadband bandpass filter. The broadband filter stage designed at 320 kHz centre frequency and 300 kHz bandwidth, the maximum gain achieved for this stage is 20 dB as the gain-bandwidth product of the utilized operational amplifier is 6.5 MHz [36]. The multiple feedback topology is selected as it is capable of achieving a high Q and gain at the same time maintain the stability. However, it provides a small amount of bandwidth [37], Figure 3a shows the circuit diagram of the filter circuit, it includes two feedback paths the first one provides low pass filter response represented by C2 and R3, and the second path provides high pass response represented by C3 and R5 [37]. Both multiple feedback bandpass filter (MFB) stages designed using staggered tuning technique [38], each stage is tuned in frequency slightly different from the 320 kHz centre frequency. The capacitor’s values are selected to be C2 and C3 are 122 pF and C4 and C5 are 100 pf, these values are tuned to reduce the effect of stray capacitors on the circuit design [39]. After choosing the capacitors, the values of the resistors are calculated based on [38].

### 3.2. Embedded Control Unit

The embedded control system is based on an MK22FN512VLH12 MCU—100 MHz Microcontroller unit (MCU) (NXP, Buckinghamshire, UK), with 512 KB flash memory, 128 KB RAM in a 64 pin LQFP package [40]. The MCU can be programmed externally with a Multilink FX programmer, via a JTAG connector. The embedded control system also contains a power management circuit (PMC) that is customized for managing the power of the system. The PMC is responsible for creating the voltage levels required by the system and delivering the power provided by a 3.7 V and 165 mAh lithium-polymer (Li-Po) battery. The 3.7 V is initially regulated down to 3.3 V by a low dropout regulator (LDO)–TLV70233DBVR—capable of supplying up to 300 mA. A 1 µF capacitor has been added to both the input and output of the device to provide as stable of an output voltage as possible. The capacitance values are suggested by the manufacturer in the datasheet of the LDO. Again, it should be noted that the embedded control unit is designed to explore the optimal communication protocol. We would envisage most of the components being implemented onto a custom ASIC in tandem with a microcontroller die in a future implantable system as per Figure 3d.

## 4. System Model and Communication Protocol

### 4.1. Frequency Analysis

**Figure 4:**
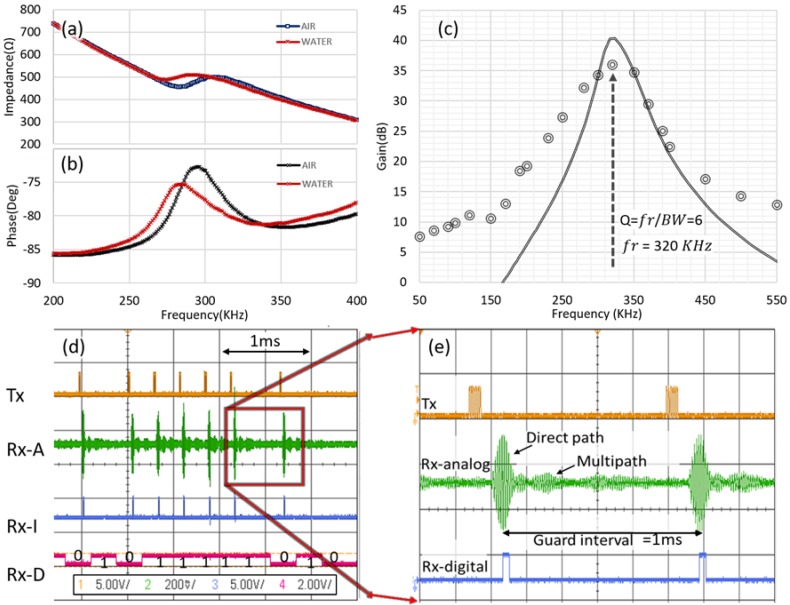
(**a**) Encapsulated transducer impedance magnitude behavior versus frequency in air and water; (**b**) Encapsulated transducer phase response versus frequency in air and water; (**c**) Simulated and measured frequency response of the designed amplifier and multiple feedback bandpass filter, the gain is 36 dB at 320 kHz and the bandwidth is 50 kHz; (**d**) Operation transient results of the proposed system, trace 1(Tx) represents the transmitted pulses at 320 kHz, trace 2(Rx-A) is the received signal after filtering the out of operation band noise and amplification and shows the multipath effect, trace 3(Rx-l) is the output of the comparator and the trace 4(Rx-D) shows the received 16 bits; (**e**) presents the direct path and multipath signals, guard interval of 1 ms inserted at the receiver to mitigate the multipath effect.

An 500 kHz MFIA impedance analyzer from Zurich Instruments (Zurich, Switzerland) was used to characterise the piezoelectric transducers. Figure 4a,b illustrate the measured impedance of the encapsulated or damped transducer in air and water respectively which provides more broadband or flat response and can run across a wide frequency range. It can be seen the real impedance value variation when changing the medium which gives an indication of good fabrication and wire bonding; therefore, the acoustic signal is not trapped in the polyurethane encapsulation wall. 

As explained earlier the receiver analog circuitry requires a precise amplification and filtration design. This circuit capable of improving the SNR performance and filter out of the band noise which makes the signal recovering at the receiver less complicated. Figure 4c illustrates the frequency response of the designed circuit, the circuit was designed using LTspice from Analog Devices (Norwood, MA, USA). The bandwidth of the measured result is 50 kHz and the achieved quality factor is 6.4 at 320 kHz cutoff frequency. However, the simulated circuit frequency response shows higher gain from the measured circuit due to the component tolerance a 4-dB gain difference between the simulated and measured results. The designed circuit consumes only 250 µA current.

### 4.2. Timing and Multipath Analysis 

We developed a test system to provide an initial basic analysis of our system. This consisted of a tank of dimensions: [50 × 30 × 25] cm to mimic a simplified human body. The transducer, pre-amp, and the filter were encapsulated in order to be safely immersed in saline. Both the transmitter and receiver were grounded with the saline in order to prevent electromagnetic cross talk.

Figure 4d illustrates the transient response of the proposed system two bytes of binary data pulsed out from the transmitter transducer at 320 kHz frequency considering the minimum delay between each consecutive pulse (guard interval = 1 ms) to avoid the surface reflected and the multipath effect signals at the receiver. OOK technique is employed to encode the binary data. It can be seen in Figure 4d the Rx-A that represents the received signal after filtration and amplification, each pulse has the primary signal (direct path) and the echo signal (multipath) that have lower amplitude from the primary signal, the multipath amplitude can be exploited to set the reference voltage input of the comparator. Rx-l signal represents the output of the comparator after comparing the received signal with the reference voltage to decide bit “1” or “0” is transmitted.

The results from Figure 4d,e show that there is a significant multipath effect (i.e., long propagation delay spread), As such a finite delay is required between each pulse which will limit the data rate. Furthermore, all systems will have a noise profile which will cause errors. As such, a communication scheme is required to ensure reliable communications. The following Table 1 lists the prototype specifications.

### 4.3. Modulation Scheme

The primary consideration from the analysis in Section 4.2 is the multipath effect and the need to provide a threshold to determine the bit-state of the signal at a specified time. There are a variety of possible modulation schemes. However, we utilized an OOK modulation scheme because of its simplicity, and robustness to the variability of different body conditions and dimensions when implanted.

In our OOK scheme the binary “1” is represented by the presence of the carrier and binary “0” is represented by the absence of the carrier signal. It can, therefore, be scalable to simple electronic hardware in future bioelectronic nodes. However, only one binary bit can be transmitted per symbol which limits the data throughput that can be achieved in this modulation scheme [41].

**Figure 5:**
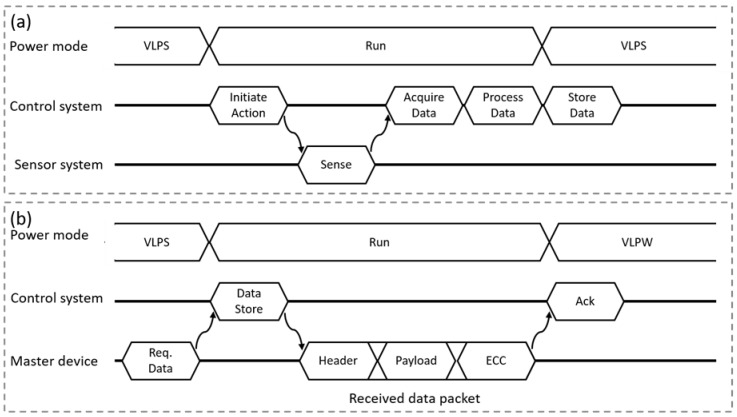
The timing diagram for the system that includes two phases (**a**) The sensor acquiring data process that starting by requesting the data from the sensor unit, the data will be processed and stored to be transmitted later; (**b**) represents the ultrasound communication link between the master and slave systems or vice versa. Power cycling is employed to reduce the current consumption by switching between the normal run and very low power modes.

Figure 5 illustrates two distinct timing diagrams, the sensing process of IMD and the ultrasound communication process respectively. The implant control system wakes up periodically to perform the sensing process. During this period, the control system initiates the sensing process. Consequently, the sensor unit starts the action. Thereafter, the control system unit implements the remaining three processes: data recording, data processing, and data storing. On the other hand, the master device sends interrupt to wake-up the implant for transmitting the stored data. Thereafter, the control system prepares the data to be transmitted by appending the overhead bits and the error correction code (ECC) to the payload. Then, the master device unit starts receiving the data which consists of three parts: header, payload and parity bits.

### 4.4. Forward Error Correction Code

We adapted a light version of the system Forward Error Correction (FEC) code to negate transmission errors. For this task, we utilized a Reed Solomon (RS) code for two reasons: (i) RS is capable of correcting burst errors since the correction occurs at the symbol level regardless of whether a signal bit or multiple bits in the symbol are in error. The symbol consists of several bits. For simplicity, in our system the symbol represents one byte, (ii) RS can be efficiently implemented in software on a microcontroller which saves the need for any additional hardware (i.e., low complexity) [42]. In addition, RS is a hard decision decoder being suitable for a low power processor, its ability to operate with non-binary symbols and it has no error floor effect so it eliminates the need for an additional Cyclic Redundancy Check (CRC) for error checking.

RS code is characterized by three main parameters *n*, k and t, *n* is the codeword that includes the payload plus parity symbols, k is the payload symbols and t is the error-correcting capability which equal t = (*n −* k)/2. In this paper, t equals 4 which means the RS code capable of correcting up to 4 bytes or symbols. The symbol size limits the codeword length (*n*), for the RS code $n=2^{m}-1$ where m is the number of bits per symbol. For 8 bits per symbol (m = 8), the maximum codeword length is 255 bytes. The payload size can be adjusted by truncating the data symbol with zero paddings but only transmitting the payload and parity bytes, and at the receiver reinserting them before passing to the decoder [43]. Table 2 summaries a comparison between three different types of codes [44]. 

### 4.5. Communication Protocol

Seeking feasibility and a balance between the design constraints and the requirements, the proposed system is a network of four bioelectronic devices nodes that are implanted inside a human body. The main function of this network is to monitor the symptoms of a particular biomarker or any other indication of any possible disorder. In addition, this network is connected to a central unit which is fixed inside the chest. The main functionalities of this unit are: controlling the process of synchronization, data communication with other bioelectronic nodes and wireless powering. Due to the harsh operation environment and the limited powering resources of the systems, there is a necessity of sketching a plan to handle the operation of messaging inside the network during its normal operation and in the emergency cases such as a sudden drop in battery level or when a test is required to check the network health status. Therefore, we proposed that the system is working by either of five modes: synchronization mode, test mode, communication mode, reporting mode, and emergency charging mode.

**Figure 6:**
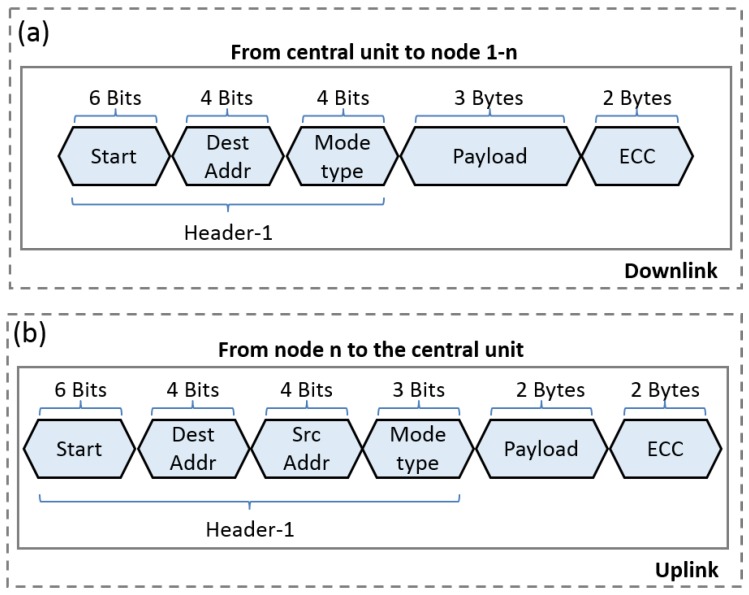
(**a**) Packet structure for the ultrasound data transmission where each node receives its packet separately. The packet includes three main parts: header, payload, and the error correction code. The downlink term represents the messaging from the central unit toward the bioelectronic nodes; (**b**) The reversed direction is referred by the uplink term.

Figure 6 shows the general packet structure, downlink, and uplink, for any type of communication mode. Both destination address and source address are referring to the network entity (bioelectronic node or the base station) unique address which is an ID of 4-bit length uploaded prior to their implantation. Moreover, the operation mode is a 4-bit field that refers to one of five modes of operations mentioned previously. Furthermore, the payload structure of each mode has its unique structure as will be described in the next section for each mode: main purpose, functionality and a brief description.

#### 4.5.1. Synchronization Mode

A synchronization scheme is essential to maintain the accuracy of data dissemination and to secure a particular time slot for each node to implement its functions. In order to prioritize the set of devices, during this mode, the central unit follows a specific procedure to announce the timeslot dedicated to each bioelectronic node. The proposed procedure is launched by the central unit via sending four messages, each one for a particular bioelectronic node in the network. As depicted in Figure 6, the message contains the address of the targeted bioelectronic node, the mode of operation, and the payload which is consisting of three parts.

The first part of the payload has a length of 3 bits and indicates the number of bioelectronic nodes in the network (n=4). The second part is of a 2 bytes length contains the addresses of the *n* bioelectronic nodes ordered in the same order of their priorities inside the network. Finally, the third part is a set of 5 bits indicating the duration of time, hereafter known as separation time ($T_{s}\leq 31\;s\;$) that are separating the window of operation of any two bioelectronic nodes has consecutive priority. In order to acknowledge the setup, a check procedure is implemented by the central unit via launching test mode as illustrated in the following section.

#### 4.5.2. Test Mode

During test mode, all devices are requested by the central unit to send a certain HELLO message after a specific duration T. Therefore, the payload field will contain two subfields: T and Hello message. Once a response is received, central unit checks whether 4 HELLO messages separated by $T_{s}$ are arrived. Consequently, synchronization of the network is acknowledged and both communication and reporting mode is ready to be launched. 

#### 4.5.3. Communication Mode

**Figure 7:**
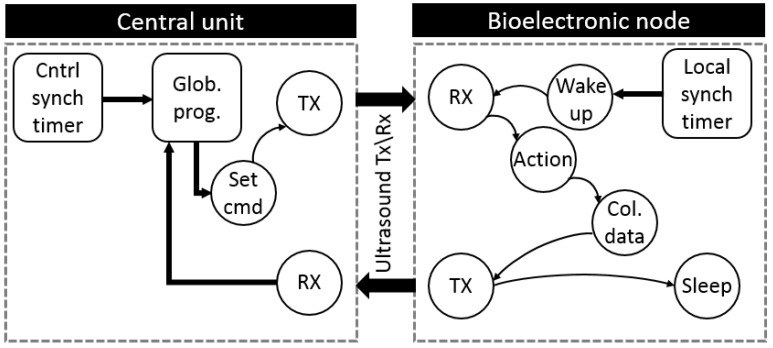
State transition diagram for the communication mode between the central unit and bioelectronic node, this mode triggered by sending a command by the central unit to the targeted bioelectronic node which replies by sending the data stored in its memory. The response time of each node is separated from other nodes by the separation time $\left(T_{s}\right)$ dedicated the synchronization mode to prevent any possible interference.

In this mode, each implant will communicate with other network entities. Such a mode is suitable to be used in case of examining the response of the biological organ, where the node is bioelectronic, to a specific treatment or stimulation. There are two types of messages in this protocol, uplink, and downlink. In the first type, a message is generated by the central unit as a request to the targeted bioelectronic node. Therefore, it consists of a destination address, operation mode and the request which is included in the payload part. As a response, an uplink message is sent from the mentioned bioelectronic node toward the requester entity. Figure 7 shows the state transition diagram for the communication mode. The utilization of intra bioelectronic nodes communication is beneficial to increases the possibility of expanding the communication coverage area by facilitating some near bioelectronic nodes to function as hops between a faraway bioelectronic node and the central unit [45]. Such a scheme requires less power consumption in comparison to expanding the coverage by increasing transmitter power.

#### 4.5.4. Reporting Mode

**Figure 8:**
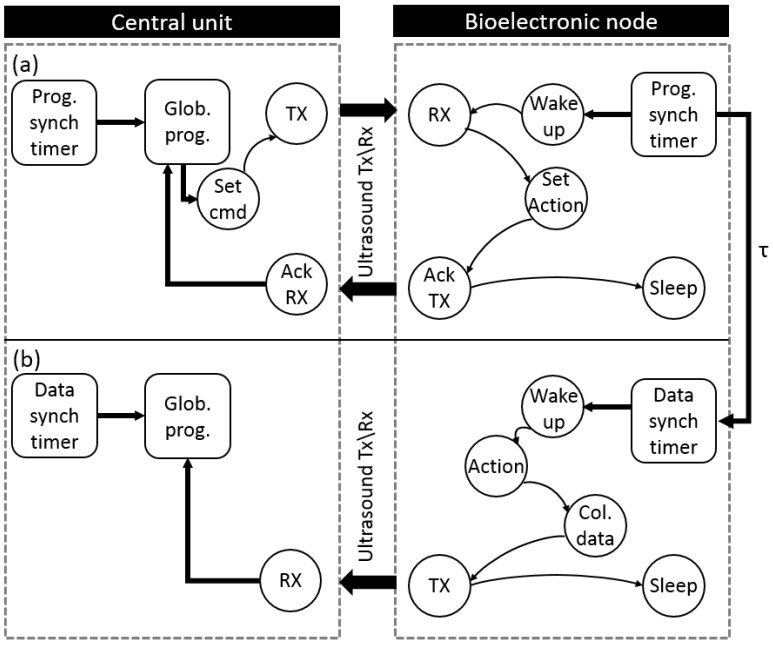
State transition diagram for the reporting mode: (**a**) depicts reporting mode initialization, where a command sent by the central unit to dedicate τ value which indicates the repetition frequency. This value is stored in the local timer; (**b**) depicts the reporting mode where the local timer wakes up the bioelectronic node every τ times. Once it wakes, the node collects data, store it and send it back to the central unit.

This mode is utilized when the bioelectronic nodes are used to collect a specific type of data frequently and send the collected data at each time. Such an operation is one of the common topologies which are governing the nowadays IMD applications such as continuous monitoring to a particular biological marker. Working in this mode does not require request generation by the central unit where data are collected and sent by the bioelectronic nodes automatically. Doing so leads to a significant reduction in power consumption where the power required to receive a request packet is saved. Launching this mode requires a presence of mode initialization as shown in Figure 8a. In this part, a downlink message is generated by the central unit and sent to the bioelectronic node in order to request a continuous reporting after a specific duration of time $T_{RS}$ dedicated by the first byte of the payload. In addition, the rest 20 bits of the payload are dedicated to identifying the repetition period (τ) which is the separating time between any two generations of the mentioned report. The bioelectronic node response, as depicted in Figure 6, is a normal communication uplink message contains both addresses of the node and the central unit are generated by the bioelectronic nodes at a frequency of $f=\;\tau^{-1}$. Figure 8b shows the second phase of operation, the local timer will wake up the node for data collection.

#### 4.5.5. Emergency Charging Mode

Due to several circumstances, a particular device might experience a sudden depletion in battery charging which is out of the design specifications and life expectancy of each battery. In such an occasion, the device launches an emergency mode by stopping all sensing operations and dedicate communication functionality to be used only to recharge its battery by using wireless acoustic signals in order to make use of the harvested power to charge its battery.

## 5. Communication System Evaluation

From our early characterization shown in Figure 4, the minimum pulse width, representing bit “1” was limited by the system bandwidth which is showed to be 50 kHz. Therefore, to achieve the maximum amplitude of the received signal, a signal of six pulse cycles are generated at 320 kHz. Pulse amplitude modulation-OOK is employed in this design, a data burst of 320 kHz is generated from the transmitter, each burst includes 16 bytes payload, bit “1” represented by six cycles (on level) and in bit “0” no pulse transmitted for the same previous period (off level). We allowed the multipath effects to decay prior to the transmission of subsequent bits by inserting a guard interval period of 1 ms. The data packet with 3.3 V amplitude is generated from the microcontroller and applied directly to the transmitter transducer to be transmitted through the channel. The receiver implant transducer receives the transmitted signal. The received signal is filtered and amplified to be applied to the threshold-based peak comparator, the reference voltage of the comparator is selected in such a way to exclude the multipath signal then extract the digital bits.

**Figure A1:**
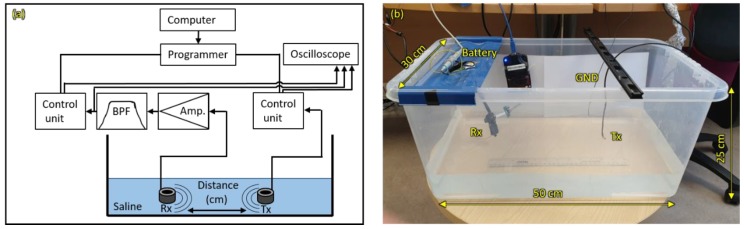
(**a**) Block diagram of the experimental set-up for the ultrasound communication link, measured results are recorded as a function of the distance between the Tx and Rx; (**b**) The experimental set-up of the communication link, tank of dimensions (50 × 30 × 25) cm is utilized for mimicking the human tissue environment. Both the transmitter and receiver are grounded with the saline in order to prevent any chance of the electromagnetic cross talk.

System evaluation was carried out at room temperature (23 °C) by placing the transmitter and receiver in the saline bath model as described in Section 4.2. We ensured that both transducers were fully submerged throughout the experiments, Appendix Figure A1 demonstrates the experimental setup of the system. In order to examine the quality of our communication link, the first test was established to measure and evaluate the SNR per pulse as a function of distance. Similar to the previously explained set-up is employed for the experiment, the RMS voltage of the received signal is measured. In order to measure the noise power, no transmission through the channel is established and the AC RMS value of the noise is measured using the oscilloscope because the noise is a random process. SNR is calculated in decibels by Equation (2), from 1 cm distance up to 30 cm:

(2) $$SNR=20\;log_{10}\left(\frac{V_{rms\;signal}}{V_{rms\;Noise}}\right)$$

The second test was established to measure the time consumed of the ultrasound pulse train when generated at the transmitter and travel through the channel till output by the receiver. The test is performed for 1 cm distance up to 30 cm at each distance the latency is measured. 

The third test was to measure and evaluate the data throughput while varying the distance for different payload sizes. The data throughput is accredited to the rate of the error-free received a packet at the receiver and calculated based on Equation (3), from 1 cm distance up to 30 cm:

(3) $$Data\;throughput=\frac{P_{L}\left(1-PER\right)}{T_{p}}$$

where $\;P_{L}$: is payload length per packet, PER: is the packet error rate and $T_{p}$: is the time spent on the data to be received at the receiver.

The fourth test is established in order to examine the performance of data transmission of our system. BER performance and packet error rate (PER) is demonstrated. The BER is calculated by dividing the number of erroneous bits by the total number of the transmitted bits, the BER presented as a function of the SNR with FEC and without FEC. The transmitted block includes 200 bits (1-byte header +16-byte payload +8-byte parity). The received payload is compared with the reference data (bit by bit) at the receiver, The BER test is run for 1000 iteration in order to achieve accurate results. The PER is calculated with and without the FEC by dividing the inappropriately received packets by the total number of the received packets, inappropriately packet represents the packets that the decoder cannot correct them but detect them. In addition, energy per bit for the microcontroller, transducer, and ultrasound electronics are calculated. The current consumption also measured for each main part of the system.

## 6. Results and Discussion

**Figure 9:**
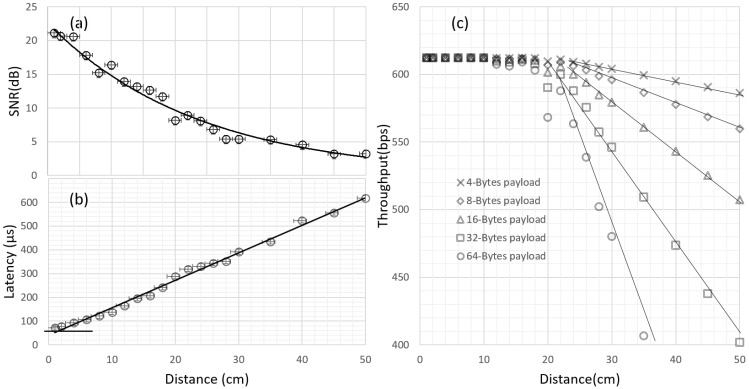
(**a**) SNR as a function of distance, a gradual decrease in SNR performance can be noticed as the distance between the Tx and Rx increases; (**b**) Latency per bit of the ultrasound signal when generated by the Tx and after it received by the RX through the channel as a function of distance; (**c**) Data throughput versus the distance between the transmitter and receiver for different payload bytes size.

Figure 9a demonstrates the SNR as a function of distance and calculated using Equation (2). A nonlinear decay in the SNR with distance can be seen. This can be easily explained in terms of the sonic wavefront expanding like a sphere over 3D space, with a spherical surface area of $\;4\pi r^{2}\;$ [46]. As such, the SNR can be expected to decrease with $1/r^{2}$. There will be additional multipath effects, but these will be temporally separated from the primary signal amplitude (which will come first). The recorded SNR = 21 dB at 1 cm distance and this value decays to 5 dB at a distance of 40 cm—approximately the distance from the chest to the bladder.

Figure 9b shows the latency of the ultrasound signal when generated at the transmitter until output by the receiver as a function of distance. It can be seen that the latency increases linearly as the distance between the implants increase, to reach 500 µs at a distance of 40 cm. This result is expected and can be calculated from the speed of sound in saline = 1540 m/s. It is nevertheless an important consideration in setting the synchronization timing between sender and receiver nodes. 

Figure 9c illustrates the data throughput as a function of distance. The highest achieved throughput is 612 bps then this value decreases as the distance increases. The ultimate limit to the data rate is determined by the guard interval (1 ms) required to negate the multipath effect. Together with overhead from headers and error correction code, this leads to a data rate of less than 1 kbit/s. 

Once the SNR drops below 10 (distance 20 cm in our measurements), errors start to present, increasing the packet error rate and thus decreasing the data rate. 

**Figure 10:**
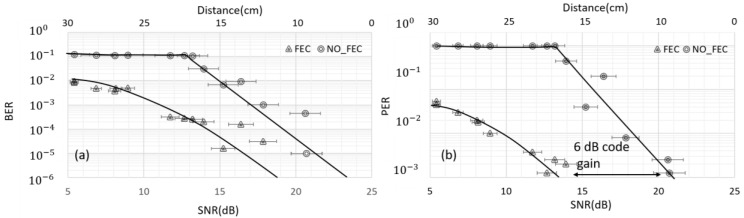
(**a**) Normalized measured BER performance of the ultrasound communication link versus the SNR for up to 30 cm distance in multipath channel, results presented with and without RS code; (**b**) Normalized measured PER versus the SNR for up to 30 cm distance in multipath channel, results presented with and without RS code.

Figure 10a demonstrates the BER performance of the ultrasound communication link without and with the RS code. It can be seen that a significant BER performance improvement in the presence of the RS code at low SNR. For instance, at 12 dB SNR the BER is $10^{-1}$ without the FEC. On the other hand, at the same SNR with the FEC the BER improved to be $10^{-3}$. This clearly demonstrates that accurate information transmission can only be achieved with error correction schemes. We feel this is highly important as commands to bioelectronics nodes need to be interpreted correctly for safe operation. Similarly, closed-loop activity needs accurate data from which to determine interventions.

Figure 10b presents the PER performance as a function of SNR and distance, it can be seen a significant enhancement of the PER at low SNR and larger distance when using FEC. For instance, at 6 dB SNR there is no way to receive an error-free packet without the FEC, whereas, for the same SNR in the presence of the FEC only 1 packet out of 100 might be received with an error. It is worth mentioning that RS code is error correction and detection which mean even if the decoded data have more than the error-correcting capability, in our case 4 bytes errors, the decoder will detect that, in this case, the packet can be rejected and the receiver can ask to resend the data again. The achieved coding gain is 6 dB with the channel coding. The achieved PER with no FEC is $10^{-3}$ at 21 dB SNR. Whereas, the same PER can be achieved at lower SNR using the FEC.

**Figure 11:**
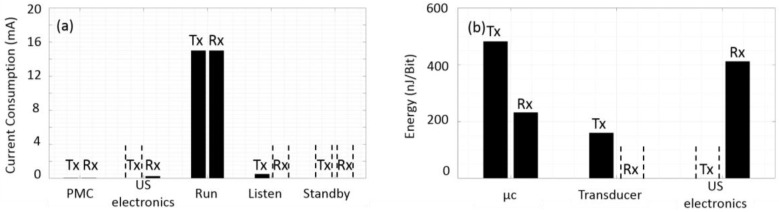
(**a**) Current consumption of both the transmitter and receiver, the PMC unit consumes 35 µA, the ultrasound electronics at the receiver only consume 250 µA, the microcontroller operates in three power modes to save the energy it consumes 15 mA, 500 µA in Tx only and 3.5 µA for the run, listen and standby modes respectively; (**b**) Energy per bit for the transmitter and receiver, the microcontroller consumes (482 and 232) nJ per bit as a transmitter and receiver respectively, the transducer energy is 160 nJ per bit and finally the receiver ultrasound electronics energy per bit 412 nJ.

Figure 11a shows the current consumption for the overall system in (mA). The current consumption of the system for the main parts that include the microcontroller in three different power cycling modes (standby, listen and run), ultrasound electronics and the power management circuit (PMC). The power management consumes 35 µA in both transmitter and receiver. The ultrasound electronics consume 250 µA, primarily to amplify the received ultrasound signal at the receiver. However, it should be noted that: (i) this power is only consumed in the periods when a signal is expected and thus the electronics are on. (ii) The ultrasound electronics were designed and implemented from off the shelf components. In a final system, the circuit current consumption and size can be significantly reduced into few µA if implemented and integrated as an ASIC chip.

From Figure 11a the Cortex-M4 microcontroller we used consumes [15, 3.5, 0.5] mA in [full, standby, listen] modes, respectively. As a general rule, microcontroller units operate most efficiently when utilized at higher speeds, followed by sleep cycling. In our case, as we operate with a timed OOK protocol, the unit can wake up, receive data, and implement any action as a result, before going back to sleep. Furthermore, the microcontroller will generate the pulses when receiving and transmitting bit “1” only then it will enter into the standby mode during bit “0” and the guard interval. Finally, it should be noted that we used the MK22FN512VLH12 microcontroller as we had prior experience with its use in rodent animal systems. However, there are lower power microcontrollers which can reduce the operating current requirement significantly. 

Figure 11b presents the energy per bit for the Tx and Rx microcontrollers, Tx transducers and Rx ultrasound electronics. It can be seen that reasonable energy can be achieved in our system during communication and processing. This result is expected for our system since it implemented with less complex receiver electronics and modulation schemes. A mathematical analysis is required to calculate the lifetime of the used battery in terms of the power budget of the proposed system. This calculation is used to compare different kinds of batteries, Based on the experimental test of our system. It was found that the transmission power consumption is equal to 18 mW and 49 mW for the ultrasound transmitter and the microcontroller in the run mode respectively as shown in Figure 10a, Consequently, the energy required to transmit one bit ($E_{b}$) can be easily calculated as: $E_{b}=P\times \;\tau$ where: τ is the duration required to send one bit. The required energy cost is 642 nJ per bit data packet.

To determine what this means in practice, the battery lifetime was calculated based on the number of packets that are required to be sent-received by the implant. The estimated lifetime of different kinds of batteries based on sending a report twice a day. On the other hand, in communication mode, battery lifetime is a function of the number of requests sent by the central unit. Reflecting the obtained results on the battery capacity and considering the three operation modes for the microcontroller and data transmission twice a day. For example, EaglePicher (Clayton, Mo, USA) provides a variety of medical grade batteries in terms of capacity and volume. For instance, a battery of 100 mAh capacity and 4.33 ${\mathrm{cm}}^{3}$ volume can last up to three and a half years based on the power consumption of each bit. Similarly, a battery of 3 mAh capacity and 0.08 ${\mathrm{cm}}^{3}$ volume can last up to forty days under the same condition. Another example is Wyon (Appenzell, Switzerland), that provides medical grade batteries with bespoke dimensions. It has miniaturized standard batteries with less capacity such as 37 mAh can last one year and three months under the same conditions that been used above to evaluate the previous types of batteries.

Taking into consideration that each bioelectronic node in the network contains an omnidirectional transducer that has a spherical coverage of a radius ρ. There are two types of topologies that can be used to govern data communication: simple stars or meshed networks. In simple star topologies, all BeNs are communicated directly with the central unit and this requires a distance which shorter than or equal to ρ. On the other hand, in the meshed network, the central unit can be far away from the bioelectronic node where the neighbor nodes can be used as relays for other BeNs. According to literature, the energy required to send each packet (transmission cost) is much lower in the meshed network in comparison with the star topology. Finally, Table 3 shows a comparison of the reported state of the art systems with our prototype.

## 7. Conclusions

We have presented a design and testing of reliable and efficient ultrasonic communication telemetry using omnidirectional transducers to implement intra body communication inside the human body. We show that over short distances, the data rate is limited by the multipath effect, which necessitated a guard interval. Over longer distances errors start to occur as the signal to noise ratio decreases. We have therefore implemented a low-overhead Reed-Solomon error correction code to ensure reliable communications.

We implemented a prototype to evaluate the system performance in saline at up to 30 cm distance between the transmitter and receiver. Short pulses sequences with guard intervals were employed to minimize the multipath effect. Error detection and correction code were implemented onto our microcontroller system to achieve reliable communication even at low SNR. Although the proposed prototype device is relatively large, it is scalable to much smaller dimensions. We have shown in Figure 3d, how it could be reduced in size to an injectable system. That would necessitate implementing our analog electronics and power management into a single ASIC chip. In addition, Further future work will be made to improve data throughput through exploration of the communication protocol and power cycling modes.

## Figures and Tables

**Table 1:** Prototype main specifications.

Parameters	Description
Prototype dimensions (mm)	(40 × 50 × 1.5)
Operating frequency (kHz)	320
US active element Shape	Soft PZT Tube
Active element dimensions (mm)	(3.5 × 2.5 × 3)
Encapsulation material	Polyurethane
Operation mode	Radial
Clock frequency	100 MHz
Application	Deep implantable Bioelectronic sensors

**Table 2:** Comparison between error detection and correction types.

Parameters	Reed Solomon Code	Cyclic Redundancy Check	Convolutional Code
Decoding type	Hard decision decoding	-	Soft decisiondecoding
Code type	Nonbinary	Binary	Binary/nonbinary
Detect and correct capability	Correct up to t symbols, where t = (*n* − k)/2	Detect burst error up to 16 bits	Limited by the constraint length (number of memory stages)
Processing	No memory requirement	Requires memory	Requires memory
Error detection and correction	Detection and correction	Detection only	Detection and correction

**Table 3:** Performance comparison of the proposed system with the state-of-the-art reported devices.

	This Work	Ref. [17]	Ref. [16]	Ref. [18]
**Frequency**	320 kHz	1.3 MHz	1 MHz	5 MHz
**Modulation**	OOK	OOK	QPSK	PPM
**Data rate**	1 Kbps	2.5 Kbps	125 Kbps	70 Kbps
**BER**	$10^{-5}$	$10^{-2}$	<$10^{-4}$	<$10^{-6}$
**Medium**	Saline	Chicken phantom	Mineral oil	Tissue phantom
**Depth**	Up to 30 cm	3 cm	5 cm	10 cm
**Energy per bit Tx and Rx**	160 nJ and 572 nJ	-	-	-
**Transducer shape**	Tube (Omnidirectional)	Sonmicrometry commercial crystal (Omnidirectional)	Plate (Directional)	Disc (Directional)
**Dimensions**	(3.5 ×2.5 × 3) mm	(1) mm	(1.08 × 1.08 × 1.44) mm	9.5 mm
